# Antibiofilm effect and mechanism of protocatechuic aldehyde against *Vibrio parahaemolyticus*

**DOI:** 10.3389/fmicb.2022.1060506

**Published:** 2022-11-10

**Authors:** Yawen Liu, Li Wang

**Affiliations:** Guangdong Provincial Key Laboratory of Food Quality and Safety, College of Food Science, South China Agricultural University, Guangzhou, China

**Keywords:** *Vibrio parahaemolyticus*, antibiofilm, protocatechuic aldehyde, comparative transcriptome analysis, bacteriostatic activity

## Abstract

This study investigated the effect of protocatechuic aldehyde (PCA) on *Vibrio parahaemolyticus* biofilm formation and its effects on gene expression. Crystal violet assay, metabolic activity assay, and fluorescence experiments were used to evaluate the antibiofilm activities of PCA and to reveal its possible antibiofilm mechanisms using transcriptomic analysis. The results indicated that the minimum antibacterial concentration of PCA against *V. parahaemolyticus* was 300 μg/mL. PCA (9.375 μg/mL) inhibited biofilm generation and adhesion of the mature biofilm. PCA (75 μg/mL) significantly reduced the metabolic viability of *V. parahaemolyticus*, reduced polysaccharide production, and inhibited cell surface flagella-mediated swimming and aggregation phenotypes. Meanwhile, transcriptome analysis showed that the key genes of *V. parahaemolyticus* expressed under PCA (75 μg/mL) inhibition were mainly related to biofilm formation (*pfkA*, *galE*, *narL*, and *oppA*), polysaccharide production and adhesion (*IF*, *fbpA*, and *yxeM*), and motility (*cheY*, *flrC*, and *fliA*). By regulating these key genes, PCA reduced biofilm formation, suppressed polysaccharide production and transport, and prevented the adhesion of *V. parahaemolyticus*, thereby reducing the virulence of *V. parahaemolyticus*. This study demonstrated that protocatechuic aldehyde can be used to control *V. parahaemolyticus* biofilm to ensure food safety.

## Introduction

*Vibrio*, found in the global marine environment, includes *V. alginolyticus*, *V. vulnificus*, and *V. parahaemolyticus* (Mok et al., 2019). Among them, *V. parahaemolyticus* has become an important pathogen of sporadic, epidemic diarrhea and food poisoning in many areas, and is increasing yearly. Some pathogenic strains of *V. parahaemolyticus* causes bacterial diseases in fish, shrimp, and shellfish, resulting in a serious economic loss (Bauer et al., 2021).

The pathogenicity of *V. parahaemolyticus* is closely related to a variety of virulence factors, including the iron absorption system, lipopolysaccharides, proteases, outer membrane proteins, the adhesion factor type III secretion system, and the type VI secretion system (He et al., 2020; Puangpee and Suanyuk, 2021). Biofilms are an important survival and pathogenic mechanism of *V. parahaemolyticus*, which are difficult to remove (Hall-Stoodley et al., 2004). Biofilms are mainly composed of proteins and polysaccharides that resist adverse environmental factors (such as UV, pH, heavy metals, and phagocytosis) and reduce sensitivity to conventional antimicrobial agents (Wang et al., 2022).

The widespread use of prophylactic antibiotics (e.g., in aquaculture) leads to increased rates of pathogen resistance, rendering many antibiotics ineffective. Cells within mature biofilms may be more resistant to antimicrobials than cells in planktonic states (Bhardwaj et al., 2021).

Therefore, new approaches are needed to treat *Vibrio* diseases or to reduce antibiotic resistance in pathogenic bacteria (Ashrafudoulla et al., 2021). Many natural compounds, including herbs, synthetic and organic plant derivatives, biosynthetic nanocomposites such as citral (Faleye et al., 2021), essential oils (Smaoui et al., 2022), blueberry extract (Sun et al., 2020), eugenol (Ashrafudoulla et al., 2020), and cationic peptide chimeras (Ning et al., 2021), have shown significant effects on biofilms. Among them, the phenols have strong antibacterial activity and have been widely verified.

PCA is a non-toxic drug excipient, preservative, and food additive that can be isolated from the water extract of *Salvia miltiorrhiza* and some as fermentation products of bacteria (Chen et al., 2021). PCA has been identified with multiple roles, including antioxidant (Guo et al., 2017), anti-inflammatory (Jieke et al., 2021), antitumor (Kyoung-Ja et al., 2008), and antimicrobial properties. Studies have found that PCA has significant effects on *Yersinia enterocolitica* (Tian et al., 2021) and *Ralstonia solanacearum* (Shili et al., 2016).

However, the antibiofilm and antivirulence activity of PCA on *V. parahaemolyticus* have not been evaluated. In this study, we explored the ability of PCA to inhibit biofilm formation and clear mature biofilms by crystal violet experiment. The inhibitory effect of PCA on the invasion and pathogenicity of *V. parahaemolyticus* was also explored by measuring bacterial motility and observing the changes in biofilm extracellular polysaccharide and bacterial numbers using Zeiss fluorescence confocal microscopy. Finally, the effect of PCA on *V. parahaemolyticus* biofilm and its possible mechanism were explored by transcriptome analysis. These results revealed for the first time the ability of PCA to inhibit the biofilm formation of *V. parahaemolyticus*, expanding the antibacterial application of PCA as a natural antioxidant food additive.

## Materials and methods

### Bacterial strains and growth conditions

*Vibrio parahaemolyticus* ATCC17802 (Guangdong Institute of Microbiology) was used as a test bacterial pathogen in this study. The cells were cultured in Tryptic Soy Broth (TSB) (3% NaCl) (Guangdong HuanKai Microbial, China) for 8 h at 37°C and then resuspended through centrifugation (2,506 × g for 10 min) in 10^8^ CFU/mL. Protocatechuic aldehyde (3,4-dihydroxybenzaldehyde, 98%) was purchased from Macklin (Shanghai, China) and the stock solutions were prepared using TSB or sterile water.

### *Vibrio parahaemolyticus* growth curves with protocatechuic aldehyde treatment

The minimum inhibitory concentrations and minimum bactericidal concentrations at different PCA concentrations were calculated using twofold dilutions. The PCA concentrations ranged from 7.8 to 1,000 μg/mL (Shili et al., 2016). The bacterial suspension was prepared using a nutrient broth containing 3% NaCl to give 10^6^ CFU/mL as a final concentration. The optical density was measured at 600 nm every 1 h for 24 h at 37°C using an automatic growth curve analyzer (Oy Growthcurves Ab Ltd.). The positive control was TSB (3% NaCl) with the bacterial suspension and without PCA. The negative control was an uninoculated TSB (3% NaCl).

### Biofilm formation inhibition assay and clearance of mature biofilms

Crystal violet staining was used to evaluate biofilm formation (Wang et al., 2022). Briefly, 100 μL of bacterial suspension (10^6^ CFU/mL) was seeded into 96-well plates with different concentrations of PCA (0, 9.375, 18.25, and 37.5 μg/mL) to form a biofilm. Inoculated or PCA-treated TSB (3% NaCl) was used as background. In addition, PCA (0, 9.375, 18.25, and 37.5 μg/mL) was added for 24 and 48 h. The scavenging effect of PCA on mature biofilms was detected as described by Qiao et al. (2021). The plate was incubated at 37°C for 24 h. Then, the plate was washed twice with sterile saline, dried for 30 min (25°C), stained with 200 μL of crystal violet (0.1% w/v), and incubated at 25°C for 20 min. Then, we washed the plate once with sterile saline. Subsequently, we added 200 μL of 33% (v/v) glacial acetic acid and measured the absorbance at 570 nm.

### Biofilm metabolic activity

*Vibrio parahaemolyticus* biofilm metabolic activity was determined using MTT (Wang et al., 2010). After forming biofilms in 96-well plates for 72 h, a PBS wash was used to remove the loosely attached cells and planktonic cells (Liu et al., 2022). To each well, thiazole blue (MTT) [3,4,5-dimethyl-2-thiazolyl]-2,5-diphenyl-2-H-tetrazolium bromide solution (5 mg/mL, prepared in sterile water) was added. The mixture was incubated at 37°C in the dark for 3 h. We then added 200 μL of dimethyl sulfoxide, incubated at 37°C for 30 min, and then measured the absorbance at 490 nm.

### Extraction and quantification of exopolysaccharides

The extracellular polysaccharides in biofilms were determined using the phenol-sulfuric acid method (Cao et al., 2021). *V. parahaemolyticus* was treated with PCA at 37°C for 24 h. The cells were centrifuged (2,500 × g for 15 min), and the supernatant was separately mixed with a threefold volume of 95% (v/v) ethanol and precipitated at 4°C for 24 h. The mixture was then centrifuged to collect the precipitates (2,500 × g for 15 min). Then, 5% phenol and 90% H_2_SO_4_ were added to the precipitate, mixed, and placed in the dark for 1 h at 25°C. The final mixture was centrifuged at 9600 × g for 10 min, and the absorbance of the supernatant at 490 nm was measured.

### Microscopy assay

The survival of *V. parahaemolyticus* under PCA inhibition was determined using a live/dead staining kit (Yu et al., 2022). Briefly, the PCA (0 and 75 μg/mL)-treated bacterial suspension was centrifuged (6,000 × g for 5 min), then 10 μL of NucGreen and EthD-III mix was added, and left in the dark for 15 min. Then the suspension was analyzed using a Zeiss fluorescence confocal microscopy (30×). Additionally, the bacterial suspension (200 μL, 10^6^ CFU/mL) and PCA solution (200 μL) were added to the 8-well chamber slides (Bhardwaj et al., 2021). The final concentrations of PCA were 75 and 300 μg/mL. The mixture was incubated at 37°C for 48 h. Then slides were washed with PBS; FITC conA (20 μg/mL) was added and stained at 25°C in the dark (30 min). Finally, the slides were washed with PBS and visualized using a Zeiss fluorescence confocal microscope (10×). The image acquisition was performed using the ZEN software. The micrographs were analyzed using the ImageJ software to evaluate the biomass and the surface volume ratio of the biofilms (Lu et al., 2021).

### Motility assay

For the swimming exercise test (Faleye et al., 2021), PCA was added to warm (45°C) TSB medium (15 mL) containing 0.3% (w/v) agar to obtain final concentrations of 0, 7.81, 15.625, 31.25, 150, and 300 μg/mL. A swarming exercise was performed using 15 mL of TSB medium containing 0.5% (w/v) agar. The plate was then dried for 1 h; then 5 μL of bacterial suspension (1 × 10^6^ CFU/mL) was added to its center and incubated at 37°C for 12 h. The diameter of the bacterial movement zone was measured using a vernier caliper (mm), and pictures were taken with Gel DOCTM XR +. A medium without PCA was used as a control.

### Transcriptome sequencing

*Vibrio parahaemolyticus* was treated with 75 μg/mL of PCA (B sample) or without PCA (A sample) as a control. Two samples of PCA were added to the logarithmic growth phase bacteria and shaken at 37°C and 150 rpm for 8 h. We extracted RNA using an RNA extraction kit (TIANGEN BIOTECH CO., Ltd.). Gene library sequencing was performed using the Illumina Hiseq platform, and quality screening was performed using FastQC (Zhang Q. et al., 2020). Reference genome alignments were performed at https://www.ncbi.nlm.nih.gov/nucleotide/CP014046.2 using Bowtie2. Differential expression analysis between the two groups of samples was performed with the DESeq2 R software package (1.16.1) [Sangon Biotech (Shanghai) Co., Ltd.] with a corrected *P*-value of 0.05 and an absolute fold change ≥ 2. Gene Ontology (GO) and Kyoto Encyclopedia of Genes and Genomes (KEGG) enrichment analyses were further profiled using the cluster Profiler package.

### Determination of gene expression using RT-qPCR

mRNA was extracted and transcribed into cDNA using an RNA extraction kit (Vazyme, Nanjing, China) and a reverse transcription kit (HiScript II Q RT Super Mix) (Vazyme) according to the kit instructions. Primer sequences (5′–3′) were designed with Primer software (Table 1). In a RT-qPCR reaction, the total volume was 20μL, which contained 0.6 μL of 10 μM of each primer F/R, 10 μL of SYBR Mix (Vazyme Biotech, Nanjing, China), 1 μL of cDNA template, and 7.8 μL of nuclease-free water. The temperature was first maintained at 95°C for 1 min in the qPCR reaction. Then, 40 cycles of temperature change at 95°C for 10 s, annealing at 55°C for 34 s, and extension at 72°C for 15 s were performed. Changes in the expression levels of target mRNAs were calculated using the 2^–△△*CT*^ method (Zhang J. et al., 2020).

**TABLE 1:** The primers for the detection of *Vibrio parahaemolyticus*.

Gene name	Sequence (5′–3′) F	Sequence (5′–3′) R
Control	TATCCTTGTTTGCCAGCGAG	CTACGACGCACTTTTTGGGA
*ectC*	CATTCTGGACAAGCACGAC	TAGTCAACGAGCGGGTAAA
*narL*	AACTCAGACGCTCTTTACG	TCTTACTGCTATTGCCTTG
*yxeM*	TGTTTGCAGACCCTTATGT	CTTTGTCGTATTGGCGTAG
*fbpA*	AACCTTGCTCGTAAACCTC	TACCCAAAGAAACATCACAT
*rpsR*	TCTTCCGTCGTCGTAAATT	GTACCAGTGATACGGCTAG

### Statistical analysis

The mean and standard deviation (SD) were calculated for triplicates. Differences between variables were tested for significance using SPSS. Standard curves and other figures were completed using Origin 2021, Graph 7, and ImageJ.

## Results

### Impact of protocatechuic aldehyde treatment on the viability of *Vibrio parahaemolyticus*

**FIGURE 1:**
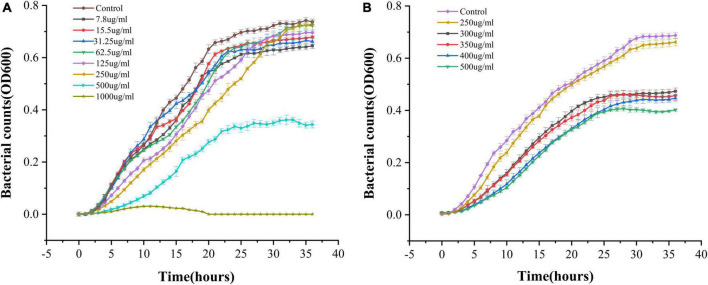
OD growth curves of *Vibrio parahaemolyticus* in the presence of different PCA concentrations at 37^°^C. **(A)** PCA concentrations of 7.8–1,000 μg/mL. **(B)** PCA concentration of 250–400 μg/mL.

The ability of PCA to inhibit the growth of *V. parahaemolyticus* was determined. With an increase in PCA concentration, the inhibitory ability of PCA against *V. parahaemolyticus* gradually increased, and the growth of *V. parahaemolyticus* almost completely stopped at 1,000 μg/mL (Figure 1). Using SPSS software for analysis, compared with the control group, PCA concentration (300 μg/mL) had a remarkable inhibitory effect on *V. parahaemolyticus*. These results indicate that PCA dose-dependently inhibited the growth of *V. parahaemolyticus*.

### Biofilm generation

**FIGURE 2:**
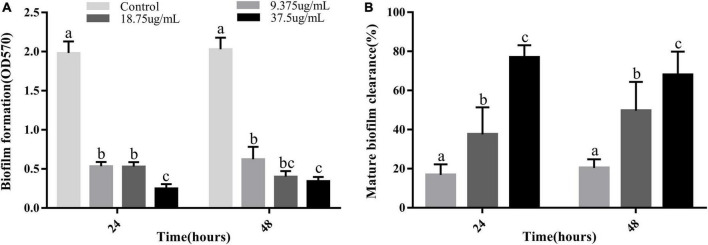
**(A)** Inhibitory effects of PCA at different concentrations on biofilm formation by *Vibrio parahaemolyticus* ATCC 17802. **(B)** Clearance of mature biofilms by different concentrations of PCA. The data are shown as means ± the Crystal violet of three independent experiments. Within each treatment, values marked with the same letter are not significantly different based on Duncan’s multiple-range test (*p* > 0.05).

*Vibrio parahaemolyticus* is a typical bacterium that forms biofilms (Bhardwaj et al., 2021). The two main methods of processing biofilms are to prevent their formation or eliminate already formed biofilms (Faleye et al., 2021). Therefore, the ability of PCA to disrupt prefabricated biofilms and its ability to clear mature biofilms was investigated. At different PCA concentrations (9.375, 18.75, and 37.5 μg/mL), the biofilm formation rate decreased by 73, 80, and 83%, respectively, after 24 h and by 69.3, 73.3, and 87.4%, respectively after 48 h (Figure 2A). Under the action of PCA (37.5 μg/mL), the clearance rates of mature biofilms at 24 and 48 h reached 76.8 and 68% (Figure 2B), respectively. We speculate that PCA not only inhibits the formation of biofilms but also considerably inhibits the adhesion of bacterial.

### Determination of the metabolic activity

**FIGURE 3:**
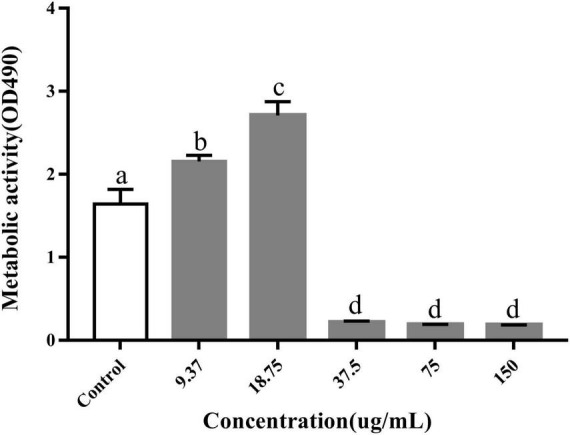
Inhibitory effects of PCA on the metabolic activity of *Vibrio parahaemolyticus* cells within biofilms. The data are shown as means ± the determination of MTT three independent experiments. Within each treatment, values marked with the same letter are not significantly different based on Duncan’s multiple-range test (*p* > 0.05).

We determined the metabolic capacity of the biofilm by measuring the metabolic activity of the cells in the biofilm (Liu et al., 2022). The intensity of the MTT releases positively correlated with cellular metabolic activity. After treatment with PCA, the cell metabolic activity increased by 23.8 and 63.5% at low PCA concentrations (9.37 and 18.75 μg/mL) (Figure 3). As shown in Figure 1, the number of bacteria did not increase at the concentration of 15.5 μg/mL compared with the control group. With an increase in PCA (75 μg/mL), the inhibitory effect on the metabolic activity of the biofilm of pathogenic bacteria increased continuously, and the metabolic activity decreased sharply by 88.59%. Then, the metabolic state of the bacteria remained stable.

### Extracellular polysaccharide assay

**FIGURE 4:**
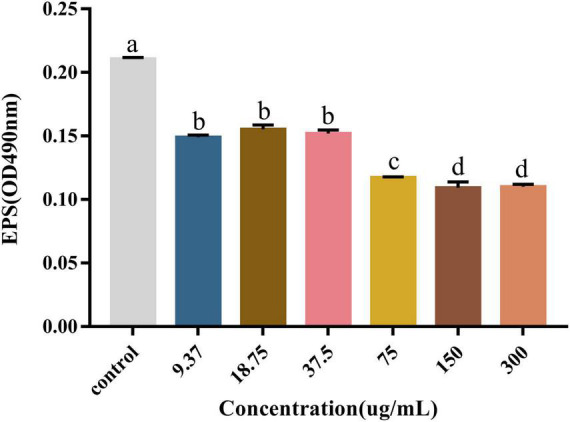
The effect of PCA on polysaccharide content in biofilm formed by *Vibrio parahaemolyticus*. The data are shown as means ± the polysaccharide three independent experiments. Within each treatment, values marked with the same letter are not significantly different based on Duncan’s multiple-range test (*p* > 0.05).

EPSs are the major part of the biofilm and directly contribute to the properties of the biofilm, especially with their strong water-binding capacity. They usually account for more than 90% of the biofilm mass (Liu et al., 2022). We used the sulfate and phenol methods to measure the polysaccharide content for evaluating the effect of PCA on EPS production. As shown, the polysaccharide content in the *V. parahaemolyticus* biofilms decreased significantly in the presence of PCA. When PCA concentration increased from 37.5 to 75 μg/mL, the polysaccharide content decreased by 27.6, 44.3, and 48% (Figure 4). The amount of biofilm formed, the biofilm metabolic activity, and the amount of polysaccharide produced decreased significantly at 75 μg/mL.

### Mobility determination

**FIGURE 5:**
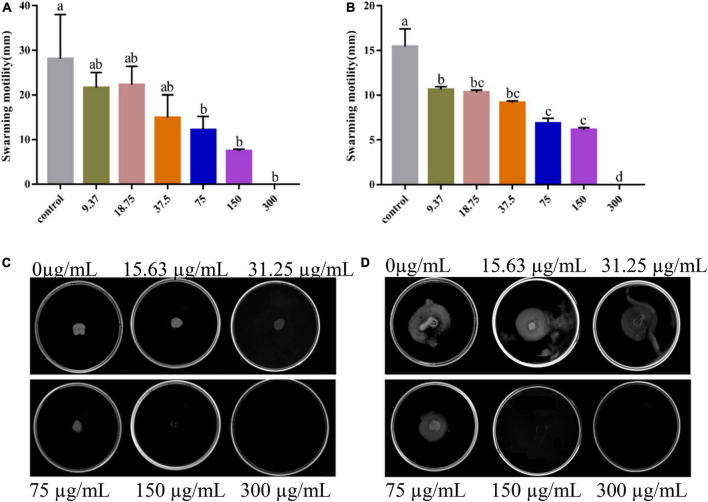
Images of swarming motility **(A)** and swimming motility **(B)** of *Vibrio parahaemolyticus* treated with PCA at different concentrations. The swarming **(C)** and swimming **(D)** areas of *Vibrio parahaemolyticus* ATCC 17802 were measured. The data are shown as means ± the motility three independent experiments. Within each treatment, values marked with the same letter are not significantly different based on Duncan’s multiple-range test (*p* > 0.05).

During the initial phase of biofilm formation, movement of *V. parahaemolyticus* is critical for the attachment to host surfaces (Faleye et al., 2021). Loss of movement may affect bacterial adhesion, impairing biofilm formation (Zhu et al., 2020). As shown, PCA remarkably inhibited the swimming and swarming ability of *V. parahaemolyticus* at 75 μg/mL concentration (Figures 5C,D). Compared with the untreated cells, the swimming Bacterial colonies area (75 and 150 μg/mL) decreased by 81.4% (Figure 5B) and 93.1%, respectively. Meanwhile, the swarming Bacterial colonies area decreased by 56.9 and 73.7% (Figure 5A), respectively.

### Microscopy inspection

**FIGURE 6:**
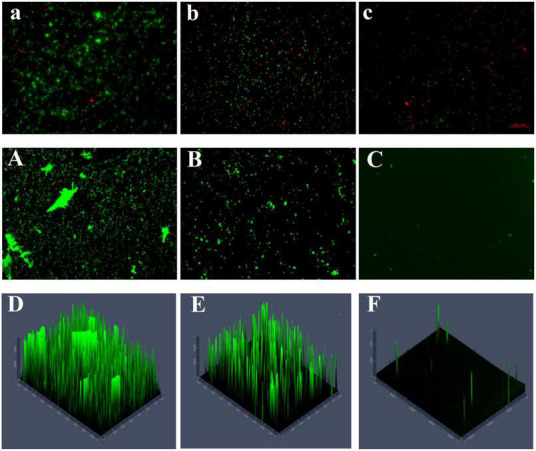
**(A)** The number of untreated dead and viable bacteria (40×). **(B,C)** The number of dead and viable bacteria treated with different PCA concentrations (75 and 150 μg/mL), green represents viable bacteria and red for dead bacteria. **(A)** The content and morphology of polysaccharides in the untreated biofilm, and the green distribution **(B,C)** indicate the polysaccharide content and morphology production in the biofilm treated under different PCA concentrations (75 and 150 μg/mL) (10×). **(D–F)** Fluorescence intensity of EPS in control and treated (75 and 150 μg/mL) biofilms.

As shown in the control group, the bacteria were wrapped in large amounts of polysaccharides, forming a large biofilm structure with a thick membrane structure (Figures 6A,D). The number of dead bacteria increased after 48 h of treating the mature biofilms with a high concentration (300 μg/mL) of PCA. In addition, biofilm formation decreased by 83.5%, and the biofilms were dispersed with a substantial reduction in thickness. The fluorescence intensity in the three-dimensional optical microscopy images changed remarkably (Figures 6C,F; Lu et al., 2021).

The number of viable bacteria was remarkably lower than that of the dead bacteria accounting for 63.7% of the total bacteria (Figure 6C). Visual results obtained with a microscope indicate that PCA reduced the biofilm of *V. parahaemolyticus* accounting for 4% (Figure 6A; Shangguan et al., 2021). The polysaccharide matrix was considerably reduced in the biofilms formed at 75 μg/mL PCA concentration. The overall membrane structure was dispersed. The biofilm area decreased by 71%, and showed low thickness variation (Figures 6B,E). The number of viable bacteria decreased by 88% under PCA (75 μg/mL) inhibition (Figure 6B).

### Transcriptome results and analysis

**FIGURE 7:**
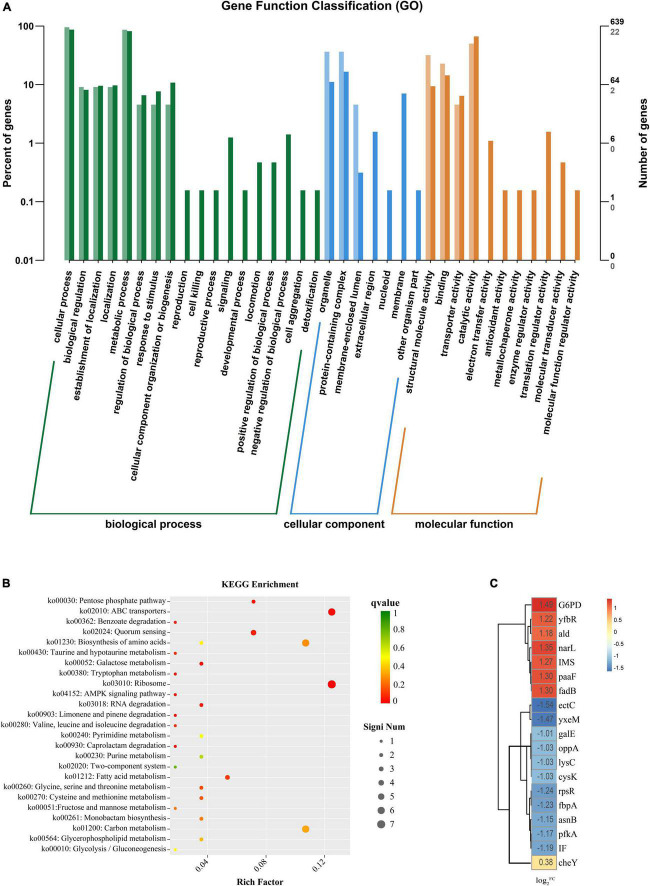
**(A)** The horizontal axis is the functional classification, and the vertical axis is the number of genes in the classification (right) and its percentage of the total number of annotated genes (left). Different colors represent different classifications. Light colors represent differential genes, and dark colors represent all genes. **(B)** The vertical axis in the figure represents the functional annotation information. The color of dots indicates the size of the *q* value, the smaller the *q* value the closer to red. The size of the dots indicates the number of differential genes included under each term, with larger points and more genes. **(C)** Heat map diagram of the log2^FC^ value of the *Vibrio parahaemolyticus* for gene. The darker the color, the larger the log2^FC^ value.

Global transcriptional analysis revealed a differential expression of 142 genes, approximately 8 h after exposure to 75 μg/mL PCA, with 63 genes upregulated and 79 genes downregulated. This mainly manifested in cell motility (Figure 7A), cell growth and death, signaling, and energy metabolism. Combined with the metabolic pathways provided by the KEGG database (Figure 7B). We found that the pathways significantly enriched in *V. parahaemolyticus* mainly included Carbohydrate metabolism, Amino acid metabolism, ABC transport process, Ribosome, Two-component system (i.e., not complete). Up-regulated or down-regulated genes are shown in Table 2.

**TABLE 2:** Top 14% genes with significant expression from RNA sequencing.

Gene ID	Gene	Log_2_ fold change	*P*-values (10^–3^)	Significant
AL464_14590	*yfbR*	1.22	0.456466	Up
AL464_09910	*narL*	1.35	10.248579	Up
AL464_03690	*paaF*	1.3	1.48 × 10^–7^	Up
AL464_03690	*fadB*	1.3	1.48 × 10^–7^	Up
AL464_11100	*G6PD*	1.49	1.78 × 10^–3^	Up
AL464_02095	*IMS*	1.27	4.86 × 10^–2^	Up
AL464_12510	*cheY*	0.375	9.9	Up
AL464_13825	*ald*	1.18	3.05 × 10^–3^	Up
AL464_05650	*pfkA*	–1.17	1.80 × 10^–10^	Down
AL464_07590	*galE*	–1.006	5.35 × 10^–2^	Down
AL464_07335	*IF*	–1.19	2.270273	Down
AL464_06250	*rpsR*	–1.24	5.72 × 10^–4^	Down
AL464_11050	*ectC*	–1.54	5.61 × 10^–10^	Down
AL464_11055	*lysC*	–1.03	1.40 × 10^–15^	Down
AL464_15200	*cysK*	–1.03	1.13 × 10^–3^	Down
AL464_15075	*asnB*	–1.15	1.21 × 10^–2^	Down
AL464_07135	*fbpA*	–1.23	0.257048	Down
AL464_03780	*yxeM*	–1.47	6.81 × 10^–3^	Down
AL464_09010	*oppA*	–1.03	8.21 × 10^–8^	Down

We analyzed the data by combining the metabolic pathways and GO annotations provided by the KEGG database. We found up-regulation of Nitrate/nitrite response regulator (*narL*) (Figure 7C) and down-regulation of ATP-dependent 6-phosphofructokinase (*pfkA*). Regulation of these two genes inhibited the expression of *frdA* and *frdB*, thereby regulating the two-component system and pentose phosphate pathway. Previous studies have found that inhibition of the bacterial two-component system (Cho and Sung-il, 2021) and pentose phosphate pathway (Kong et al., 2022) may promote carbohydrate intake and dephosphorylation of the phosphotransferase system. Kong et al. (2022) found that the inhibition of the pentose phosphate pathway may reduce the intracellular accumulation of carbohydrates such as glucose, thereby inhibiting bacterial exopolysaccharide synthesis and biofilm formation; Meanwhile, it may also reduce the efficiency of glucose transport, thereby destroying the energy metabolism of bacteria and inhibiting its cell viability.

After PCA was applied to *V. parahaemolyticus*, glycolysis and TCA cycle were also involved in the internal regulation of the bacteria. The up-regulation of isopropyl malate synthase (*IMS*) and alanine dehydrogenase (*ald*) affect acetyl-CoA formation, thereby regulating glycolysis and TCA cycle. In addition, the up-regulation of transcription factor glucose-6-phosphate 1 dehydrogenase (*G6PD*) promotes the conversion of NADP to NADPH. Tan et al. (2022) found that after antibiotic inhibition, internal carbon and nitrogen metabolism in *V. parahaemolyticus* is involved in the activation of bacterial glycolysis and TCA cycle to promote ATP accumulation, and the increase of NADPH maintains a stable cellular state and also promotes amino acid metabolism to enhance antibiotic tolerance. At the same time, we found that enoyl-CoA hydratase (*paaF*) and 3-hydroxyalkyl-CoA dehydrogenase (*fadB*) were up-regulated in *V. parahaemolyticus*, which may lead to enhanced tryptophan or β-alanine metabolism. Dukes et al. (2015) found that by promoting tryptophan or β-alanine metabolism, producing pyruvate and acetyl-CoA to activate mTOR, it would promote glycolysis and TCA cycle. However, this promotion of glycolysis and the TCA cycle by pyruvate and acetyl-CoA reaches a threshold (Yang et al., 2020).

The metabolism of amino acids in *V. parahaemolyticus* also changes after PCA inhibition. The down-regulation of L-ectoine synthase (*ectC*), aspartate kinase (*lysC*), cysteine synthetase (*cysK*), and asparagine synthetase (*asnB*) related genes involved in amino acid synthesis (Figure 7C) may result in the decreased amino acid synthesis of aspartic acid, glutamic acid, threonine, and cysteine. Down-regulation of small subunit ribosomal protein S18 (*rpsR*) also directly affects amino acid substitutions. In ribosomes, the small subunit ribosomal protein S15 (*IF*) responsible for adhesion and invasion is downregulated. At the same time, the iron (III) transport system substrate-binding protein (*fbpA*) and putative amino acid ABC transporter substrate-binding protein (*yxeM*) were also significantly down-regulated. In biofilm proteomics of *V. parahaemolyticus*, Guo et al. (2020) found that increased glutamate and threonine promoted the synthesis of extracellular proteins in biofilms and down-regulation of *cysK* gene may regulate the secretion of toxins by bacteria to inhibit the growth of neighboring cells. Zhu et al. (2020) found in their proteomic studies on *V. parahaemolyticus* that the ABC transport system is closely related to bacterial adhesion ability. Thereby down-regulation of genes related to the ABC transport system may hinder material transport and reduce bacterial adhesion and biofilm formation.

In addition, under the inhibition of PCA, the chemotaxis receptor response regulator (*cheY*) in the internal genes of *V. parahaemolyticus* was down-regulated, and *cheY* could directly inhibit Flageller motor switch adaptation. A gene associated with bacterial quorum sensing, oligopeptide transport system substrate-binding protein (*oppA*), was down-regulated. Upregulation of 5’-deoxynucleotides (*yfbR*) associated with eDNA synthesis in biofilms is upregulated. This can lead to reduced motility, adhesion, and biofilm production (Kong et al., 2020). Chang et al. (2020) found that *cheY* could modulate Flageller motor in bacteria, thereby affecting bacterial motility. Sun et al. (2022) hypothesized that quorum sensing may be involved in regulating pilus production to control biofilm generation. Renfei et al. (2019) and Wu et al. (2022) found that quorum-sensing regulators affect gene transcription in the lateral flagella of *V. parahaemolyticus* to regulate swarming movement. In general, PCA inhibits the biofilm of *V. parahaemolyticus* by regulating a variety of genes.

### Validation of RNA-seq data by RT-qPCR

We screened biofilm-related genes by high-throughput sequencing and verified the reliability of the data by RT-qPCR. Four significantly decreased genes and one significantly increased gene were screened. The gene *narL* related to bacterial metabolism was up-regulated. The relative expression of this gene with values of 217% of the control group (Figure 3). The ABC transport system genes *fbpA* and *yxeM* related to biofilm clearance and production were down-regulated. *rpsR* gene related to amino acid synthesis was down-regulated. The bacterial growth-related gene *ectC* was down-regulated. The relative expression of these genes was significantly reduced with values of 1.4%, 13.5, 3.7, and 7.5% of the control group, respectively. These results demonstrated that 75 μg/mL PCA could effectively inhibit the biofilm formation of *V. parahaemolyticus*.

## Discussion

It is important to control *V. parahaemolyticus* in the global outbreak of diseases and contaminated food. In recent years, the antibacterial ability of many natural antibacterial substances has been investigated. Cinnamaldehyde and some of its derivatives have been found to inhibit biofilm formation and motility, and quorum sensing-related virulence gene expression in *V. parahaemolyticus* (Faleye et al., 2021). Citral can effectively inhibit the adhesion ability and flagellar biosynthesis of *V. parahaemolyticus* (Yi et al., 2019). Tea polyphenols have been found to reduce the immune capacity of *V. parahaemolyticus* and improve the vibrio resistance of shrimp (Qin et al., 2021). As a kind of polyphenol, the application of PCA effectively reduced the incidence of bacterial wilt, and the control effect was up to 92.01% after 9 days of inoculation (Shili et al., 2016). Tian et al. (2021) showed that PCA could cause morphological changes in *Yersinia enterocolitica*, destroy intracellular ATP and pH, and significantly inhibit the growth of the bacteria. However, the inhibitory effect of PCA on *V. parahaemolyticus* has not been studied. We investigated the inhibitory effect of PCA on *V. parahaemolyticus* biofilm and its mechanism.

The results showed that the polysaccharide content was significantly reduced by 48%, the biofilm clearance rate reached 78% (Figure 4), and the biofilm thickness became increasingly thinner (Figures 6D–F), and moved from a large structure to a dispersed structure (Figures 6A–C). Probably through inhibition of carbohydrate uptake and phosphorylation (*pfkA*, *galE*, and *narL*) (Kong et al., 2020), PCA reduces exopolysaccharide secretion in *V. parahaemolyticus*, affecting bacterial surface adhesion and virulence (Hwang et al., 2012). At the same time, PCA inhibits biofilm adhesion and reduces biofilm formation by inhibiting *IF* expression and polysaccharide production or transport. In addition, PCA may also reduce the accumulation of cysteine and aspartate by regulating the expression of *ectC*, *lysC*, *cysK*, and *asnB*. Upregulation of *yfbR* may lead to reduced eDNA synthesis (Kong et al., 2020), promoting the self-organization of biofilm structural communities and promoting cell-to-cell gene transmission (Brown et al., 2015). Downregulation of *oppA* directly inhibits biofilm formation, promotes bacterial necrotivity, and stops the production of degradative enzymes (Sun et al., 2022).

At low concentrations of PCA (9.37 and 18.75 μg/mL), The metabolic activity of *V. parahaemolyticus* increased by 23.8 and 63.5%, respectively, in metabolic activity (Figure 3). But the bacterial numbers did not increase at these concentrations. PCA may regulate transcription factors, such as *paaF*, *fadB*, *IMS*, and *ald*, causing ATP accumulation. *G6PD* also increases NADPH and reduces bacterial stress response (Dukes et al., 2015; Gong et al., 2020). However, owing to the negative feedback regulation of the signaling pathway in bacteria, the metabolic activity decreased rapidly at 37.5 μg/mL PCA concentration (Figure 3). Some studies have also reported that cinnamaldehyde and eugenol significantly inhibited bacterial adhesion ability and metabolic activity (Liu et al., 2022).

**FIGURE 8:**
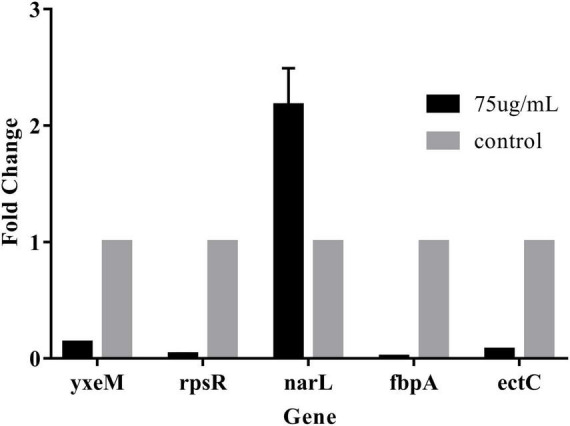
The gene expression levels were determined by RT-qPCR. The 2^–△△CT^ method was applied to determine the expression levels using 16S as the control gene. The data are shown as means ± the RT-qPCR three independent experiments. Within each treatment, values marked with the same letter are not significantly different based on Duncan’s multiple-range test (*p* > 0.05).

We found that PCA suppresses the expression of class III flagellar system genes and biofilm formation by regulating the transcription factors *cheY*, *flrC*, and *fliA* (Li et al., 2022), thereby inhibiting Vibrio motility (McCarter, 2001). As the concentration of PCA increases, the bacterial colony area decreased, and the motility capacity of *V. parahaemolyticus* is almost lost at the 150 μg/mL PCA concentration (Figures 5B,D). Some substances have similar effects on bacterial motility and downregulate related genes, such as thymoquinone (Guo et al., 2019). Other virulence factors of *V. parahaemolyticus* also change under PCA inhibition, including downregulation of the *oppA*, regulation of a transcription factor involved in quorum sensing, and reduction in the adhesion of the virulence factor T6SS2 to host cells (Wu et al., 2022). In addition, PCA significantly inhibits the *fbpA* and *yxeM* genes, affecting material transport, and reducing biofilm formation (Figure 8; Gregory et al., 2020).

Transcriptional regulation high-throughput analysis revealed that PCA mainly regulates related downstream genes. We speculate that PCA may activate the bacterial signaling system, thereby inducing transcriptional changes in downstream genes.

## Conclusion

In this study, we demonstrated that 75 μg/mL of PCA had potent antibiofilm properties against *V. parahaemolyticus*. At a PCA concentration of 37.5 μg/mL, biofilm production was greatly reduced (Figure 2A). Meanwhile, PCA regulated the expression of biofilm-related genes *oppA*, *fbpA*, and *yxeM*. With the increase of concentration, PCA greatly inhibited the metabolism of *V. parahaemolyticus* (Figure 3) and up-regulated the expression of *G6PD*, *ald*, *paaF*, and *fadB*. In addition, PCA decreased the production of exopolysaccharides and regulated the expression of related genes *pfkA*, *galE*, and *IF*. The clearance rate of *V. parahaemolyticus* increased with the increase in PCA concentration. The motility of *V. parahaemolyticus* was also dose-dependent on the concentration of PCA. Taken together, PCA significant inhibited the biofilm formation, adhesion and motility of *V. parahaemolyticus*.

Therefore, PCA may be developed as commercially more efficient and safer antimicrobial additives to curb *V. parahaemolyticus* biofilms in food systems and to alleviate foodborne diseases caused by such pathogens, which is of great importance in ensuring food safety.

## Data availability statement

The original contributions presented in the study are included in the article/supplementary material, further inquiries can be directed to the corresponding author.

## Author contributions

YL was responsible for the conception and filling of the manuscript. LW was responsible for the review and feasibility analysis of the manuscript. Both authors contributed to the article and approved the submitted version.

## References

[B1] Ashrafudoulla M. Mizan M. F. R. Ha A. J. Park S. H. Ha S. D. (2020). Antibacterial and antibiofilm mechanism of eugenol against antibiotic resistance *Vibrio parahaemolyticus*. *Food Microbiol.* 91:103500. 10.1016/j.fm.2020.103500 32539983

[B2] Ashrafudoulla M. Na K. W. M. Hossain I. Mizan M. F. R. Nahar S. Toushik S. H. (2021). Molecular and pathogenic characterization of *Vibrio parahaemolyticus* isolated from seafood. *Mar. Pollut. Bull.* 172:112927. 10.1016/j.marpolbul.2021.112927 34526263

[B3] Bauer J. Teitge F. Neffe L. Adamek M. Jung A. Peppler C. (2021). Impact of a reduced water salinity on the composition of *Vibrio* spp. in recirculating aquaculture systems for Pacific white shrimp (*Litopenaeus vannamei*) and its possible risks for shrimp health and food safety. *J. Fish Dis.* 44 89–105. 10.1111/jfd.13270 32971569

[B4] Bhardwaj D. K. Taneja N. K. Dp S. Chakotiya A. Patel P. Taneja P. (2021). Phenotypic and genotypic characterization of biofilm forming, antimicrobial resistant, pathogenic *Escherichia coli* isolated from Indian dairy and meat products. *Int. J. Food Microbiol.* 336:108899. 10.1016/j.ijfoodmicro.2020.108899 33160121

[B5] Brown H. L. Kate H. Mark R. Roy P. B. Arnoud H. M. V. (2015). Campylobacter jejuni biofilms contain extracellular DNA and are sensitive to DNase I treatment. *Front. Microbiol.* 6:699. 10.3389/fmicb.2015.00699 26217328 PMC4498105

[B6] Cao J. Liu H. Wang Y. He X. Jiang H. Yao J. (2021). Antimicrobial and antivirulence efficacies of citral against foodborne pathogen *Vibrio parahaemolyticus* RIMD2210633. *Food Control* 120:107507. 10.1016/j.foodcont.2020.107507

[B7] Chang Y. J. Zhang K. Carroll B. L. Zhao X. W. Charon N. W. Norris S. J. (2020). Molecular mechanism for rotational switching of the bacterial flagellar motor. *Nat. Struct. Mol. Biol.* 27 1041–1047. 10.1038/s41594-020-0497-2 32895555 PMC8129871

[B8] Chen X. Yuan M. Wang Y. Zhou Y. Sun X. (2021). Influence of fermentation with different lactic acid bacteria and *in vitro* digestion on the change of phenolic compounds in fermented kiwifruit pulps. *Int. J. Food Sci. Technol.* 57 2670–2679. 10.1111/ijfs.15316

[B9] Cho S. Y. Sung-il Y. (2021). Structural analysis of the activation and DNA interactions of the response regulator VbrR from *Vibrio parahaemolyticus*. *Biochem. Biophys. Res. Commun.* 555 102–108. 10.1016/j.bbrc.2021.03.114 33813268

[B10] Dukes A. Davis C. Refaey E. M. Upadhyay S. Mork S. Arounleut P. (2015). The aromatic amino acid tryptophan stimulates skeletal muscle IGF1/p70s6k/mTor signaling *in vivo* and the expression of myogenic genes *in vitro*. *Nutrition* 31 1018–1024. 10.1016/j.nut.2015.02.011 26059377 PMC4465076

[B11] Faleye O. S. Sathiyamoorthi E. Lee J. H. Lee J. (2021). Inhibitory effects of cinnamaldehyde derivatives on biofilm formation and virulence factors in *Vibrio* species. *Pharmaceutics* 13:2176. 10.3390/pharmaceutics13122176 34959457 PMC8708114

[B12] Gong Q. Y. Yang M. J. Yang L. F. Chen Z. G. Jiang M. Peng B. (2020). Metabolic modulation of redox state confounds fish survival against *Vibrio alginolyticus* infection. *Microb. Biotechnol.* 13 796–812. 10.1111/1751-7915.13553 32212318 PMC7664012

[B13] Gregory G. J. Dutta A. Parashar V. Boyd E. F. (2020). Investigations of dimethylglycine, glycine betaine, and ectoine uptake by a betaine-carnitine-choline transporter family transporter with diverse substrate specificity in *Vibrio* species. *J. Bacteriol.* 202 314–320. 10.1128/jb.00314-20 32817090 PMC7685552

[B14] Guo C. Wang S. Duan J. Jia N. Zhu Y. Ding Y. (2017). Protocatechuic aldehyde protects against cerebral ischemia-reperfusion-induced oxidative injury via protein kinase cepsilon/Nrf2/HO-1 pathway. *Mol. Neurobiol.* 54 833–845. 10.1007/s12035-016-9690-z 26780453

[B15] Guo D. Yang Z. Zheng X. Kang S. Yang Z. Xu Y. (2019). Thymoquinone inhibits biofilm formation and attachment-invasion in host cells of *Vibrio parahaemolyticus*. *Foodborne Pathog. Dis.* 16 671–678. 10.1089/fpd.2018.2591 31070474

[B16] Guo L. X. Wang J. J. Yi G. Ling T. Haiquan L. Yingjie P. (2020). Comparative proteomics reveals stress responses of *Vibrio parahaemolyticus* biofilm on different surfaces: Internal adaptation and external adjustment. *Sci. Total Environ.* 731:138386. 10.1016/j.scitotenv.2020.138386 32417469

[B17] Hall-Stoodley L. Costerton J. W. Stoodley P. (2004). Bacterial biofilms: From the natural environment to infectious diseases. *Nat. Rev. Microbiol.* 2 95–108. 10.1038/nrmicro821 15040259

[B18] He Y. U. Wang S. Yin X. Sun F. He B. Liu X. (2020). Comparison of extracellular proteins from virulent and avirulent *Vibrio parahaemolyticus* strains to identify potential virulence factors. *J. Food Prot.* 83 155–162. 10.4315/0362-028x.jfp-19-188 31860395

[B19] Hwang G. Kang S. El-Din M. G. Liu Y. (2012). Impact of an extracellular polymeric substance (EPS) precoating on the initial adhesion of *Burkholderia cepacia* and *Pseudomonas aeruginosa*. *Biofouling* 28 525–538. 10.1080/08927014.2012.694138 22686692

[B20] Jieke Y. Jianchun L. Ruizhi T. Xingcan H. Xiao L. Xia Z. (2021). Protocatechuic aldehyde attenuates obstructive nephropathy through inhibiting lncRNA9884 induced inflammation. *Phytother. Res.* 35 1521–1533. 10.1002/ptr.6919 33118280

[B21] Kong T. Lin S. Ren X. Li S. Gong Y. (2020). Transcriptome and metabolome integration analysis of mud crab Scylla paramamosain challenged to *Vibrio parahaemolyticus* infection. *Fish Shellfish Immunol.* 103 430–437. 10.1016/j.fsi.2020.05.069 32473364

[B22] Kong X. Li C. Sun X. Niu B. Guo D. Jiang Y. (2022). The maltose transporter subunit IICB of the phosphotransferase system: An important factor for biofilm formation of *Cronobacter*. *Int. J. Food Microbiol.* 370:109517. 10.1016/j.ijfoodmicro.2021.109517 35216827

[B23] Kyoung-Ja K. Mi-Ae K. Jee-Hyung J. (2008). Antitumor and antioxidant activity of protocatechuic aldehyde produced from *Streptomyces lincolnensis* M-20. *Arch. Pharm. Res.* 31 1572–1577. 10.1007/s12272-001-2153-7 19099226

[B24] Li L. Lu J. Zhan P. Qiu Q. Chen J. Xiong J. (2022). RNA-seq analysis unveils temperature and nutrient adaptation mechanisms relevant for pathogenicity in *Vibrio parahaemolyticus*. *Aquaculture* 558:738397. 10.1016/j.aquaculture.2022.738397

[B25] Liu H. Zhu W. Cao Y. Gao J. Jin T. Qin N. (2022). Punicalagin inhibits biofilm formation and virulence gene expression of *Vibrio parahaemolyticus*. *Food Control* 139:109045. 10.1016/j.foodcont.2022.109045

[B26] Lu C. Liu H. Shangguan W. Chen S. Zhong Q. (2021). Antibiofilm activities of the cinnamon extract against i and *Escherichia coli*. *Arch. Microbiol.* 203 125–135. 10.1007/s00203-020-02008-5 32772125

[B27] McCarter L. L. (2001). Polar flagellar motility of the *Vibrionaceae*. *Microbiol. Mol. Biol. Rev.* 65 445–462. 10.1128/mmbr.65.3.445-462.2001 11528005 PMC99036

[B28] Mok J. S. Ryu A. Kwon J. Y. Park K. Shim K. B. (2019). Abundance, antimicrobial resistance, and virulence of pathogenic *Vibrio* strains from molluscan shellfish farms along the Korean coast. *Mar. Pollut. Bull.* 149:1105590. 10.1016/j.marpolbul.2019.110559 31543492

[B29] Ning H. Cong Y. Lin H. Wang J. (2021). Development of cationic peptide chimeric lysins based on phage lysin Lysqdvp001 and their antibacterial effects against *Vibrio parahaemolyticus*: A preliminary study. *Int. J. Food Microbiol.* 358:109396. 10.1016/j.ijfoodmicro.2021.109396 34560361

[B30] Puangpee S. Suanyuk N. (2021). *In vitro* and *in vivo* evaluation of antimicrobial activity of Zooshikella marina against pathogenic bacteria causing *vibriosis* in aquaculture. *Aquac. Res.* 52 4996–5007. 10.1111/are.15371

[B31] Qiao Y. Jia R. Luo Y. Feng L. (2021). The inhibitory effect of Ulva fasciata on culturability, motility, and biofilm formation of *Vibrio parahaemolyticus* ATCC17802. *Int. Microbiol.* 24 301–310. 10.1007/s10123-021-00165-1 33638013

[B32] Qin G. Chunyan Z. Qingguo C. Jiahui C. Hui C. (2021). Synergistic inhibition effects of tea polyphenols as adjuvant of oxytetracycline on *Vibrio parahaemolyticus* and enhancement of *Vibriosis* resistance of *Exopalaemon carinicauda*. *Aquac. Res.* 52 3900–3910. 10.1111/are.15234

[B33] Renfei L. Hao T. Yue Q. Wenhui Y. Huiying Y. Dongsheng Z. (2019). Quorum sensing regulates the transcription of lateral flagellar genes in *Vibrio parahaemolyticus*. *Future Microbiol.* 50 795–810. 10.2217/fmb-2019-0048 31469011

[B34] Shangguan W. Xie T. Zhang R. Lu C. Han X. Zhong Q. (2021). Anti-biofilm potential of kefir-derived *Lactobacillus paracasei* L10 against *Vibrio parahaemolyticus*. *Lett. Appl. Microbiol.* 73 750–758. 10.1111/lam.13568 34586634

[B35] Shili L. Yanmei Y. Juanni C. Bing G. Liang Y. Wei D. (2016). Evaluation of the antibacterial effects and mechanism of action of protocatechuic aldehyde against *Ralstonia solanacearum*. *Molecules* 21:754. 27294898 10.3390/molecules21060754 PMC6274444

[B36] Smaoui S. Hlima H. B. Tavares L. Ennouri K. Braiek O. B. Mellouli L. (2022). Application of essential oils in meat packaging: A systemic review of recent literature. *Food Control* 132:108566. 10.1016/j.foodcont.2021.108566

[B37] Sun J. Li X. Qiu Y. Xue X. Zhang M. Yang W. (2022). Quorum sensing regulates transcription of the pilin gene mshA1 of MSHA pilus in *Vibrio parahaemolyticus*. *Gene* 807:145961. 10.1016/j.gene.2021.145961 34530088

[B38] Sun X. Hao L. Xie Q. Lan W. Zhao Y. Pan Y. (2020). Antimicrobial effects and membrane damage mechanism of blueberry (*Vaccinium corymbosum* L) extract against *Vibrio parahaemolyticus*. *Food Control* 1111:107020.

[B39] Tan X. Qiao J. Wang J. L. Li H. D. Wang X. Y. (2022). Characterization of ampicillin-resistant genes in *Vibrio parahaemolyticus*. *Microb. Pathog.* 168:105573. 10.1016/j.micpath.2022.105573 35588966

[B40] Tian L. Wang X. Zhang D. Wu M. Xue Z. Liu Z. (2021). Evaluation of the membrane damage mechanism of protocatechuic aldehyde against Yersinia enterocolitica and simulation of growth inhibition in pork. *Food Chem.* 363:130340. 10.1016/j.foodchem.2021.130340 34144416

[B41] Wang H. Cheng H. Wang F. Wei D. Wang X. (2010). An improved 3-(4,5-dimethylthiazol-2-yl)-2, 5-diphenyl tetrazolium bromide (MTT) reduction assay for evaluating the viability of *Escherichia coli* cells. *J. Microbiol. Methods* 82 330–333. 10.1016/j.mimet.2010.06.014 20619304

[B42] Wang H. Zou H. Wang Y. Jin J. Wang H. Zhou M. (2022). Inhibition effect of epigallocatechin gallate on the growth and biofilm formation of *Vibrio parahaemolyticus*. *Lett. Appl. Microbiol.* 5 81–88. 10.1111/lam.13712 35353911

[B43] Wu K. Long Y. Liu Q. Wang W. Fan G. Long H. (2022). CqsA-introduced quorum sensing inhibits type VI secretion system 2 through an OpaR-dependent pathway in *Vibrio parahaemolyticus*. *Microb. Pathog.* 162:105334. 10.1016/j.micpath.2021.105334 34915139

[B44] Yang M. J. Xu D. Yang D. X. Li L. Peng X. X. Chen Z. G. (2020). Malate enhances survival of zebrafish against *Vibrio alginolyticus* infection in the same manner as taurine. *Virulence* 11 349–364. 10.1080/21505594.2020.1750123 32316833 PMC7199751

[B45] Yi S. Du G. Zi H. Huihui S. Zhanwen Z. Xiaodong X. (2019). Attenuation of multiple *Vibrio parahaemolyticus* virulence factors by citral. *Front. Microbiol.* 10:894. 10.3389/fmicb.2019.00894 31073298 PMC6495081

[B46] Yu H. Pei J. Qiu W. Mei J. Xie J. (2022). The antimicrobial effect of *Melissa officinalis* L. Essential oil on *Vibrio parahaemolyticus*: Insights based on the cell membrane and external structure. *Front. Microbiol.* 13:812792. 10.3389/fmicb.2022.812792 35359730 PMC8961409

[B47] Zhang J. Wang L. Shi L. Chen X. Chen C. Hong Z. (2020). Survival strategy of *Cronobacter sakazakii* against ampicillin pressure: Induction of the viable but nonculturable state. *Int. J. Food Microbiol.* 334:108819. 10.1016/j.ijfoodmicro.2020.108819 32818765

[B48] Zhang Q. Wang L. Liu Z. Zhao Z. Zhao J. Wang Z. (2020). Transcriptome and metabolome profiling unveil the mechanisms of *Ziziphus jujuba* mill. peel coloration. *Food Chem.* 312:125903. 10.1016/j.foodchem.2019.125903 31901700

[B49] Zhu Z. Yang L. Yu P. Wang Y. Peng X. Chen L. (2020). Comparative proteomics and secretomics revealed virulence and antibiotic resistance-associated factors in *Vibrio parahaemolyticus* recovered from commonly consumed aquatic products. *Front. Microbiol.* 11:1453. 10.3389/fmicb.2020.01453 32765437 PMC7381183

